# Hydration and Dehydration Prevention in Nursing Homes: Perspectives, Barriers, and Practices of Care Teams and Managers

**DOI:** 10.3390/nu18040630

**Published:** 2026-02-14

**Authors:** Elena Paraíso-Pueyo, Cristina Vallès-Carvajal, Carla Camí, Teresa Botigué, Laia Selva-Pareja, Rosa Mar Alzuria-Alós

**Affiliations:** 1 Department of Nursing and Physiotherapy, University of Lleida, Montserrat Roig St., 2, 25198 Lleida, Spain; elena.paraiso@udl.cat (E.P.-P.); cristina.valles@udl.cat (C.V.-C.); teresa.botigue@udl.cat (T.B.); laia.selva@udl.cat (L.S.-P.); rosamar.alzuria@udl.cat (R.M.A.-A.); 2 Health Education, Nursing, Sustainability and Innovation Research Group (GREISI), 25198 Lleida, Spain; 3 Lleida Institute for Biomedical Research, Dr. Pifarré Foundation, IRB Lleida, 25198 Lleida, Spain; 4 Chair of Development of Healthy and Sustainable Organizations and Territories (DOTSS), University of Lleida, 25001 Lleida, Spain

**Keywords:** dehydration, hydration management, long-term care, nursing homes, nursing staff, older adults

## Abstract

**Background:** Low-intake dehydration is frequent among institutionalised older adults and is associated with high morbidity–mortality and healthcare costs. Its prevention requires effective strategies and professional and institutional coordination. **Objective**: This study aims to explore the knowledge on the identification and prevention of dehydration, as well as the management of hydration by healthcare professionals and management in a nursing home. **Methods**: This exploratory qualitative study with a phenomenological approach convened two focus groups with 18 nurses and assistants alongside two semi-structured interviews with managers. The content analysis addressed five dimensions: knowledge; identification of dehydration; prevention of dehydration; barriers and facilitators; and actions proposed to improve hydration. **Results**: Participants recognised the importance of hydration but reported barriers including limited training, absence of specific protocols, and imprecise record systems. Facilitators included hydration reminders, improved accessibility to water, sensorial resources, promotion of independence, social activities, and institutional support for preventive strategies. **Conclusions**: These findings show that preventing and managing dehydration in nursing homes is complex and can be influenced by organisational and structural factors. The nursing team plays a central role in detecting dehydration early and implementing personalised strategies to promote fluid intake, while managerial support strengthens their effectiveness. Improving staff training, developing practical guidelines, and refining record systems may help address the identified barriers and enhance person-centred hydration management aligned with residents’ needs.

## 1. Introduction

Older adults have a higher risk of low-intake dehydration due to physiological changes associated with ageing, as well as a decrease in the feeling of thirst and a deterioration of renal function [1,2,3,4,5,6]. In addition, this risk can increase due to the presence of comorbidities, polypharmacy, and physical and mental disability [6,7,8]. This, along with the ageing population, leads to an increase in the number of individuals who are susceptible to dehydration [9]. Although this situation is less frequent among older adults who live alone, it affects more than a third of those with a higher degree of fragility or dependence, particularly among those who are institutionalised in nursing homes [4,10].

The prevalence of low-intake dehydration in nursing homes oscillates between 0.8% and 38.5%, depending on the biochemical markers of dehydration utilised [3,5,11]. A recent systematic review [5] estimated a prevalence of 26% based on serum or plasma osmolality as the reference method. In particular, a study conducted in a Spanish nursing home [12] reported that 34% of the residents drank less than 1500 mL/d, the minimum recommended level for water intake, and up to 79.2% drank less than the calculated Skipper standard [13], which is considered the most efficient method for individualising hydration adjusted to weight [14]. These findings reflect a concerning situation, as dehydration in this population group is associated with an increase in mortality, unfavourable clinical change, and higher healthcare costs [6,9,11,15].

Despite its significant frequency and repercussion in institutionalised individuals, low-intake dehydration continues to be a prevalent problem in nursing homes [6,16]. The staff, due to their direct and constant contact with the residents, play a fundamental role in its prevention and management, as they can implement strategies that favour adequate hydration [17,18]. Nevertheless, institutional and professional barriers exist that make the efficient management of hydration somewhat difficult [18,19].

Even though comprehensive approaches to prevent and address hydration exist [17,18,20], most of the qualitative studies conducted in nursing homes have only included some of the agents involved [21,22,23]. These studies focused on different professional profiles which limits the cross-cutting understanding of the care provided. Some focused on older adults and nursing staff [21,24], while others included occupational therapy assistants and hospitality staff [22], as well as dietitians and medical records personnel [23]. Although some studies included managers [17,25,26], two of them [25,26] primarily focused on the qualitative evaluation of a specific intervention. These studies did perform an in-depth analysis of how management decisions interact with frontline practice, but they did not discuss the synergies and divergences between care staff and managers. Consequently, there is little evidence showing the organisational frictions, communication gaps, and role-specific priorities that can influence hydration care at all levels.

Addressing this gap requires qualitative approaches that combine multiple professional perspectives. This would help to elucidate how care is delivered in daily practice, and it would also help to understand how care is coordinated and what constraints it may face. Integrating the perspectives of nurses, nursing assistants, and managers would offer a more comprehensive understanding of the factors that influence hydration care. In addition, it would shed light on the dynamics that remain invisible in studies that have solely focused on a single professional group.

For this, the aim of the present study is to explore the knowledge of healthcare professionals and managers in a nursing home in the identification, prevention, and management of dehydration, and help guarantee the adequate hydration of the residents.

## 2. Materials and Methods

### 2.1. Study Design

A qualitative exploratory study was conducted using a descriptive phenomenological approach. This approach allowed the development of a comprehensive and corroborated understanding of hydration and its management, particularly from the perspective of the professionals involved in the daily care of residents and the management of the centre.

### 2.2. Context and Participants

The study was conducted in a nursing home in Lleida (Spain).

The sample was obtained through convenience sampling for healthcare professionals and purposive sampling for the centre managers, with the latter limited to the two existing management positions. Participation was voluntary in all cases.

The inclusion criteria for the healthcare professionals were nurses or nursing assistants who provided hydration care to the residents and who had worked for a minimum of 2 years at the centre studied.

### 2.3. Instruments and Data Collection

The information was obtained by conducting two focus groups with healthcare professionals, as well as two semi-structured interviews directed to two centre managers. These took place at the nursing home in a comfortable and quiet space, which allowed the participants to freely express their opinions and preserved the confidentiality of the information shared.

Both focus groups comprised 8 and 10 healthcare professionals, respectively. The planning of the groups was agreed upon between the professionals, managers, and researchers in order to minimise interference with the normal workday and to ease the participation of professionals in accordance with their work shifts. Each focus group session lasted around 60 min and was conducted by two study researchers (CC, CV). Both focus groups were audio recorded and complemented with the information recorded into a field notebook during the sessions.

In addition, the researchers RMA and EP conducted semi-structured interviews with each of the centre’s managers, which lasted between 40 and 60 min. Likewise, both were recorded and complemented with individual observations.

A guide with open-ended questions was used to explore the perception of both the professionals and the managers on the knowledge, identification, prevention, and management of dehydration, along with the collaboration in the management and improvement of water intake (Table 1).

### 2.4. Data Analysis and Interpretation of Results

The focus groups and interviews were transcribed verbatim and analysed using ATLAS.ti 25 software (ATLAS.ti Scientific Software Development GmbH, Berlin, Germany) following an inductive approach. Two researchers independently coded the transcripts, generating initial descriptive codes from which a preliminary codebook was inductively developed and refined throughout the analysis. Coding discrepancies were discussed in consensus meetings with a third researcher until agreement was reached. Data saturation was considered reached when no new codes or categories emerged in the final interviews and focus groups. Through this iterative process, initial codes were grouped into broader categories that captured the core themes that emerged from the data. The final themes were synthesised into a results report and returned to participants for verification.

During the study, compliance with the criteria of scientific rigour proposed by Graneheim et al. [27] was guaranteed, including credibility, dependence, and transferability. To conduct and evaluate the study, the COREQ verification for qualitative designs was used [28].

### 2.5. Ethical Approval

Approval was obtained from the Research Ethics Committee for drugs at the Arnau de Vilanova University Hospital, Lleida Territorial Management—GSS, Spain (CEIC-3054, 22 April 2024). All the participants were previously informed about the details of the study, underlining the voluntary character of their participation. Likewise, they were explicitly asked to provide their consent before participating.

## 3. Results

A total of 18 professionals participated in the focus groups conducted with healthcare professionals. The age range oscillated between 20 and 63 years old, most were women (94.4%), and 83.3% were nursing assistants. On average, the participants had 7 years of experience in the area of geriatrics and more than 4 years working at the centre studied (Table 2).

The semi-structured interviews were given to two managers, both of whom were women. Given the limited sample size, the characteristics of the sample were not detailed in order to avoid their identification and to guarantee their anonymity.

The findings from the present study were grouped into five dimensions that emerged from the analyses performed: (1) knowledge about dehydration; (2) identification of dehydration; (3) prevention of dehydration; (4) addressing barriers and facilitators to hydration; and (5) actions suggested by the healthcare team to improve hydration. In the provided verbatim transcriptions, HCG1 denotes Healthcare Professionals Group 1, HCG2 denotes Healthcare Professionals Group 2, and M denotes Managers.

### 3.1. Knowledge About Dehydration

The healthcare professionals conceptualised dehydration as a lack of water in the body, associated it with physiological changes due to ageing, and recognised signs such as a decrease in urine volume and skin changes:

*“Lack of water… in the body.” (HCG1-1)*

*“With age, the feeling or desire to drink water decreases…” (HCG2-1)*

*“…In the diaper you can see that it’s dark…” (HCG1-5); “…he is urinating little, if he doesn’t drink.” (HCG1-3)*

On their part, the managers highlighted the impact of dehydration on the health of the residents and the increase in hospital referrals due to it:

*“…It involves changes in health status, especially…” (M1)*

*“Increased hospital referrals due to worsening health status of dehydrated residents.” (M2)*

In both cases, healthcare professionals and managers recognised the lack of specific information on dehydration, in both their professional training and posterior refresher courses:

*“We lack more knowledge about dehydration.” (HCG1-5)*

*“I remember there was nothing specific about hydration… it would be an important aspect to study it in the degree.” (M1)*

### 3.2. Identification of Dehydration

The managers stated that the nurses and nursing assistants were responsible for detecting dehydration signs through observation and examination. In addition, an annual blood test was given that included sodium (Na) and potassium (K) levels:

*“…They are the ones who spend seven hours with them on the floor… they are the ones who warn us: I don’t see him well… then it’s assessed…” (M1)*

*“They can detect it in their day-to-day… but the situation must be very obvious.” (M2)*

*“…They look at the condition of the skin, to see if it’s hydrated…” (M1)*

*“…Sometimes they tell us: the urine is very dark; he is probably dehydrated.” (M1)*

*“When we do the annual assessments… there is a specific analysis regarding Na, K levels…” (M1)*

However, both healthcare professionals and managers recognised that dehydration was not a priority when assessing residents:

*“We don’t give much importance to dehydration, to be honest.” (HCG1-7)*

*“…at the moment when, if a resident falls ill or there is some pathology, I think it is the last thing that is looked at or not taken into account.” (M1)*

### 3.3. Prevention of Dehydration

The healthcare professionals considered that dehydration prevention was crucial due to its impact on the physical and mental health of the residents:

*“…It’s important because, if not, many things will start to fail.” (HCG1-6)*

*“It’s important to prevent dehydration for the mental and physical health of the user.” (HCG2-4)*

On their part, the managers underlined the importance of maintaining homeostasis to avoid hospital referrals:

*“…Hydration is very important, as it can sometimes save you from having to go to the emergency room.” (M1)*

Both the healthcare professionals and the managers indicated that dehydration prevention was addressed through established care routines, such as programmed water rounds and personalisation according to the needs and history of each resident:

*“…We have water rounds… the assistants have a little more time to dedicate to the hydration of the adults, they have it very ingrained… they know that after the activity, a glass of juice, or water for everyone, and if it’s hot, we give them more…” (M1)*

*“…It’s important to know the history of each person, because if they didn’t drink water at home, and they come to the centre: I’m going to tell them to drink water, and if they have never drunk it… their history is also important…” (HCG2-3)*

In addition, the managers described how, in hot weather, water intake increased:

*“…especially in hot weather, it’s when we insist more on drinking water…” (M1)*

### 3.4. Addressing Barriers and Facilitators to Hydration

Both groups of informants pointed out that the logistic management of hydration was the responsibility of the nursing team, collaborating closely with the kitchen staff to guarantee an efficient and continuous source of water. Nevertheless, the healthcare professionals identified it as a limitation when proposing a greater accessibility to water:

*“Generally, you ask nursing, but if it’s not there, then you go to the kitchen and they take it up to the floor…” (HCG1-10)*

*“That residents can have, as well as us, easier access to water …” (HCG1-5)*

To promote and facilitate hydration, reminders directed to the residents about the importance of hydration were underlined, especially during heat waves. In addition, during the hottest seasons, they also advised about the use of additional resources, such as juice or refrigerators, to facilitate hydration:

*“They remind them that it will be very hot: above all, drink lots of water…” (M1)*

*“…He doesn’t like the water, but instead the peach juice, which is a little thicker, with a little thickener, he drinks it very well.” (HCG1-5)*

*“…we bought new refrigerators with greater capacity, so that each resident can have fresh water… this way we can have a small bottle in the refrigerator for each resident…” (M2)*

On the one hand, the healthcare professionals stated that some residents responded better to medical indications, while other residents resisted drinking due to their habit of low fluid intake. On the other hand, individuals with cognitive deterioration often did not recognise the need to drink, nor could they express it adequately:

*“…if the doctor says it, they listen to you.” (HCG1-10)*

*“…they say to you: And why should I drink, if I have never drunk in my life!” (HCG1-10)*

*“…he doesn’t even realise he needs water. He doesn’t know how to express it.” (HCG2-3)*

Another aspect that emerged was related to the monitoring and recording of water intake. Informants explained that the recording system used in the institution was designed to only indicate whether the resident had drunk or not without documenting the amount consumed. However, one manager indicated that this was not always recorded. In this sense, the healthcare professionals expressed that recording intakes was an excessive workload, which negatively affected resident care:

*“…the only thing that is not recorded is the amount taken…” (HCG1-10)*

*“…I check it and I say to them: haven’t you recorded it? … It’s no use to me if they tell me: yes, he has drunk everything, if it’s not recorded…” (M1)*

*“…Anything that involves sitting at a computer recording data takes away care…” (M2)*

Lastly, both groups of informants underlined good communication and collaboration, pointing out key aspects such as the organisation between shifts and the weekly meetings to adequately coordinate patient care:

*“We have very good communication.” (HCG1-3, HCG1-4, HCG1-10)*

*“We organise between shifts, if there’s anything, we talk to each other.” (HCG2-1)*

*“We have weekly meetings with the entire team and if there has been something, it can be discussed there.” (M1)*

Nevertheless, both identified difficulties in the transfer of information between shifts and the lack of specific protocols, which limited a more efficient approach:

*“As a specific dehydration protocol, there is none… the water intakes are scheduled, and they know that they are at 9, at 13, at 17…” (M1)*

### 3.5. Actions Suggested by the Healthcare Team to Improve Hydration

Among the actions to improve hydration, the managers suggested the implementation of specific hydration protocols and the use of reminders through public address systems and signage. To improve the accessibility of water for residents, they proposed the use of jugs available at every floor or the installation of water fountains. They also underlined the need for staff training and the improvement and proactivity of the team:

*“Develop a specific protocol, with information that reinforces the importance of hydration.” (M1)*

*“Inform them over the PA system: “time for hydration.” The more independent residents and the assistants would hear it and take the necessary measures: it’s my turn to drink…” (M1)*

*“Reminder signs could also be put up: drink water.” (M1)*

*“…installing fountains would be very useful… it would give a lot of access to those who are more independent.” (M2)*

*“…teach them about the importance of the resident being well hydrated.” (M1)*

*“Feedback between professionals is important; being proactive depends on each person’s involvement.” (M1)*

On the other hand, the actions proposed by healthcare professionals were centred on adapting the flavour of the drinks, as some residents did not accept the current flavours provided or the use of thickeners, while juices were better accepted. In addition, they also proposed sensorial support such as relaxing water sounds. And lastly, they proposed direct actions with their residents, such as using direct reminders, training, promoting independence, and promoting empathic conversations to reinforce awareness about the importance of hydration:

*“They don’t like the taste of the thickener.” (HCG1-2)*

*“…and nobody leaves the juice…they drink it…” (HCG2-5)*

*“…Sometimes we play relaxation music… we could put something about water therapy.” (HCG2-1)*

*“Remind them that they have water within reach, and that they can drink it.” (HCG2-7)*

*“A workshop, talk or activity could be held for residents to improve their water intake.” (HCG1-3)*

*“…through an empathic conversation with the person.” (HCG2-3)*

*“Give them more independence, so they can drink water at will… otherwise you should be giving them water…” (HCG2-7)*

## 4. Discussion

The present study explored the knowledge of healthcare professionals and managers on dehydration at a nursing home, indicating that although both groups recognised its importance, they faced organisational and training barriers. By analysing these two professional levels together, the study combined complementary perspectives and identified areas for improvement in coordination and organisational tensions. It also highlighted the specific priorities of each role, which have not been apparent in studies that focused on a single group. The coordination of the team, with nursing playing a main role, and the implementation of personalised strategies were noted as key facilitators for comprehensive care.

Both healthcare professionals and managers indicated having limited knowledge about dehydration, coinciding with the need for specific training, in agreement with other studies [17,24,29]. However, they were still able to define dehydration and identify the evident signs and clinical symptoms. Nevertheless, these signs shouldn’t be used in an isolated manner, given their limited diagnostic evidence, in accordance with current recommendations [4,30]. Due to this, combining them with an individual assessment of the resident and blood analysis parameters could improve diagnostic precision [30]. Presently, an annual blood test is performed, including Na and K concentrations, but this does not consider serum osmolarity, the gold standard in low-intake dehydration diagnosis, according to current clinical guidelines and expert consensus [4,30,31]. This gap could indicate that current practices only partially align with clinical hydration assessment frameworks, thereby limiting their practical application. In contrast, the managers showed knowledge that was more centred on the institutional dimension of hydration, assessing its impact based on quality indicators such as referrals to emergencies or hospital admittances, but they were dependent on the healthcare staff for the direct detection of the clinical signs. These divergences in responsibilities and approaches could suggest that while both groups have some knowledge gaps in common, they have distinct yet complementary competency profiles. This complementarity emphasises the importance of examining both roles together. Consequently, training programmes should be designed to suit the specific roles of each group, with a focus on reinforcing the clinical assessment skills of healthcare personnel and strengthening the organisational and supervisory competencies of managers. This would ensure that training can respond more effectively to the decision-making needs and specific roles of each group.

The early detection of dehydration was mainly the responsibility of nurses and nursing assistants, as they were in close contact with the residents and could apply direct observation strategies. However, our findings showed that routine assessments did not systematically prioritise hydration. Consistent with the evidence, this often leads to late detection when the clinical consequences are already evident [4,6]. This lack of prioritisation was sometimes reinforced by the automation of routines, such as water-intake rounds, which could generate a false sense of compliance when hydration was assumed rather than actively verified [17].

In addition, the recording systems focused on what was supplied and not what was consumed, making precise monitoring more difficult as described in other studies [18,25,26,32] and reflecting a low ability to assess the real intake [25,32,33,34]. The managers supervised these processes and results, although their direct participation in the observation was limited. This distribution of roles underlined that the effectiveness of the detection depended on the coordination between healthcare professionals and managers [35]. In this regard, more accurate methods for recording the intakes of fluids could help to overcome current limitations, whether through simplified records or digital tools [26,32]. However, their implementation must not increase staff workload, as this has already been identified as an obstacle in the literature [18]. Therefore, future research should examine how these tools can be feasibly integrated into routine practice to improve monitoring of fluid intake. Taken together, these factors highlight a critical gap in risk awareness that contributes to delays in early identification and could have significant clinical consequences.

In this context, the nursing team should play a central role, as nurses usually take the lead in the implementation and supervision of preventive strategies [18,24,25,31]. Their work could ensure the consistent integration of these measures in daily care while promoting the involvement of those in charge of management at the same time, as their collaboration has shown to increase the viability and compliance of the strategies [4,35,36,37].

The prevention of dehydration was implemented through scheduled fluid-intake rounds, adapted to the preferences of each resident, and integrated into the daily routine of the staff, coinciding with the current recommendations [4,5,9,25,38]. Both healthcare professionals and managers coincided when pointing out the importance of these measures, albeit from different perspectives. The healthcare professionals focused their attention on the personalisation of the care, the adaptation of textures and flavours, the use of sensorial supports, and the promotion of hydration for residents through empathetic support. These findings were consistent with the evidence, which points to the efficacy of these strategies in increasing intake [4,5,6,9,17,18,21,36]. On their part, the managers took on a centre role in organisational planning, the implementation of specific strategies, and the supervision of institutional results, as well as intensifying these measures in extreme weather conditions. This distribution of responsibilities reflected the clear differentiation of the approaches: the healthcare professionals prioritise the health of the residents [17,22,39] while the managers tended to focus on quality indicators and organisational efficiency [39]. Thus, the effective prevention of dehydration will require combining both perspectives to guarantee that individualised care can be supported by structured management [24,35]. The joint awareness of healthcare professionals and managers regarding the comprehensive dimension of hydration is key for implementing coherent and sustainable strategies [17,37].

Among the barriers identified, the resistance of some residents to the intake of liquids due to low consumption habits or lack of thirst stood out in line with previous evidence [6,17,21,24]. In addition, the residents with cognitive deterioration did not recognise the need to drink or could not express it, thus requiring active support from staff. Lastly, the limitations of the recording systems associated with the workload and the additional time required, with the consequent reduction in the direct care of the residents, were also described. This situation may be related with structural factors such as care overload [21] and the low nurse-to-patient ratio in Spain [40], becoming a significant barrier for adequate support in hydration [18,21]. The managers, on their part, recognised these difficulties from the areas of planning and supervision, highlighting the need to optimise resources and the organisation of the team. Effective coordination, communication between shifts, and periodic meetings emerged as key facilitators for guaranteeing the adequate intake of fluids, as pointed out by Lea et al. [22] and Mentes et al. [41].

In this scenario, the nursing team has a key role in the early detection and prevention of dehydration, as nurses lead interdisciplinary coordination and supervise the implementation of individualised care routines [17,18,24,25,29]. Their leadership balances the direct care of healthcare professionals with the planning of managers, favouring the implementation of protocols [4] and promoting evidence-based decisions [17,21,22], such as visual and audio reminders [41] as well as awareness activities for residents [18,42]. All of these can reinforce the safety and quality of care for residents [17].

Looking to the future, it is recommended that structured strategies are implemented and directed to both groups. These include specific periodic training on the early detection of dehydration, clinical reasoning, and the comprehensive assessment of a person’s hydration state [4,24,29]. In addition, improvements should be made with recording systems, protocols, and strategies centred on the person, such as visual and audio reminders, sensorial stimuli, diversification of flavours, and the promotion of independence [18,21,22,23,25]. The active coordination between healthcare professionals and managers could strengthen the effectiveness of these strategies, promoting adherence to the hydration routines, reducing health risks, and optimising the indicators of institutional quality [22,43]. Therefore, the combination of training, organisation, and personalised care can be comprehensive approach that guarantees adequate hydration in nursing homes [17,29,35].

Although the study was conducted in a single centre, several identified aspects, such as work organisation, coordination between roles, difficulties in recording hydration, and the predominance of nursing leadership, have also been common in other long-term care centres with similar structures, resident profiles, and care models. These aspects could therefore be transferable to comparable contexts.

### Limitations

The study was conducted in a single nursing home, which may limit the transferability of the findings. Nevertheless, it can offer a valuable context for continuing to explore the perceptions of both the healthcare team and the centre’s management. In addition, the sample of managers and nurses was limited to the positions within the centre. Even so, the inclusion of the managers could provide additional insights to the results, especially regarding institutional factors. In addition, as this was a Spanish nursing home, the results reflected the health and work characteristics found in Spain, though they could still be relevant in similar settings.

Another limitation is related to the sampling process. Nurses and nursing assistants were recruited through convenience sampling, while managers were selected through opinion sampling. These approaches may introduce a selection bias, limiting the representativeness of the sample. Voluntary participation could favour the involvement of more motivated or aware professionals, potentially excluding those with different perspectives.

A further limitation was the absence of the perspectives of older people under institutional care. Their participation could have enriched the understanding of hydration management from a person-centred care approach. However, this was not possible due to the clinical and functional profile of residents within long-term care facilities in Spain. These residents were characterised by high levels of cognitive impairment and dependency, as reported by Botigué et al. [12]. Such conditions may hinder meaningful participation in qualitative interviews and could give rise to ethical and methodological issues.

Finally, since no direct observation of the practice was carried out, the results were based solely on the participants’ own accounts. This issue may have influenced the results, either by underestimating routine omissions or overestimating compliance with hydration practices, given that self-reported data are susceptible to recall bias and social desirability effects. These considerations should be taken into account when interpreting the consistency and accuracy of the reported practices. Additionally, although strategies to enhance rigour were applied, such as independent coding, consensus meetings, and participant validation, researchers’ positionality may still have influenced the interpretation, as is common in qualitative phenomenological research.

## 5. Conclusions

The findings show the complexity of preventing, detecting, and addressing dehydration in nursing homes, as it is influenced by institutional, organisational, and structural factors. In this sense, the nursing team emerged as a key player in the early detection of dehydration and the implementation of personalised strategies to promote the adequate intake of fluids, while the support from management was essential for the success of these actions. In addition, the need for specific training was evidenced, as well as practical guidelines and more precise recording systems to guide the staff and guarantee the adequate monitoring of hydration. Likewise, sensorial resources, reminders, access to water, the promotion of independence, and social activities were identified as facilitating practices that could favour a more effective management centred on the person. Based on the current findings, new lines of research emerge to address the limitations identified, with priority given to understanding hydration care through the inclusion of residents’ own perspectives in line with a person-centred care approach. In addition, multi-centre and mixed-methods studies are needed to combine qualitative insights with objective clinical indicators and strengthen the evidence base. These approaches may support the development of nursing-led interventions aimed at promoting safe, humanised, and person-centred hydration care in long-term care settings.

## Figures and Tables

**Table 1:** Topics and examples of prompts for focus groups and semi-structured interviews.

Topic	Example Prompts Included	
Knowledge and training on dehydration	FG	What do you know about dehydration in older people?
	S-SI	Do you think healthcare professionals have sufficient knowledge about this condition?
Identification and assessment of dehydration	FG	How do you detect dehydration in residents?
	S-SI	Do you have protocols to prevent and detect dehydration?
Time spent on hydration	FG	How do you manage your workday to ensure hydration?
	S-SI	Do you think they dedicate sufficient time during their workday to address this need?
Resources and facilitators for adequate hydration	FG	How do you rate the resources available for monitoring hydration? What resources could help improve water intake?
	S-SI	What resources would be useful for monitoring this condition?
Barriers to hydration management	FG	What obstacles hinder adequate hydration of residents?
	S-SI	What logistical challenges might limit residents’ access to water? And what challenges might professionals face?
Inter-professional coordination and institutional management	FG	How is hydration managed during shift changes to ensure continuity of care? How do you assess the centre’s efforts to manage hydration?
	S-SI	What improvements do you think could be implemented to improve hydration management?

FG: Focus group; S-SI: Semi-structured interviews.

**Table 2:** Sociodemographic characteristics of the healthcare professionals.

Characteristics		n (%)
Gender	Men	1 (5.6)
	Women	17 (94.4)
	Non-binary/Other	0 (0.0)
Country of origin	Dominican Republic	1 (5.6)
	Honduras	1 (5.6)
	Peru	1 (5.6)
	Romania	2 (11.1)
	Spain	12 (66.7)
	Venezuela	1 (5.6)
Occupation	Nurse	3 (16.7)
	Nurse assistant	15 (83.3)
		**mean ± SD**
Age		38.7 ± 14.5
Years worked in the area of geriatrics		7.4 ± 5.5
Length of time at the centre		4.6 ± 2.4

SD: Standard deviation.

## Data Availability

As the present study was conducted in the city of Lleida, which has a relatively small population, making the data openly available would entail a high risk of participant re-identification. Specifically, the combination of the geographical context and the nature of the responses could make it possible to identify individual participants, thereby posing an ethical risk and breaching the confidentiality assurances provided. For this reason, and in accordance with the conditions approved by the relevant ethics committee, the data cannot be shared in open-access repositories or made publicly available.

## Abbreviations

The following abbreviations are used in this manuscript:

FG	Focus group
S-SI	Semi-structured interviews
